# Effect of Polymer Permeability and Solvent Removal Rate on *In Situ* Forming Implants: Drug Burst Release and Microstructure

**DOI:** 10.3390/pharmaceutics11100520

**Published:** 2019-10-10

**Authors:** Xiaowei Zhang, Liqun Yang, Chong Zhang, Danhua Liu, Shu Meng, Wei Zhang, Shengnan Meng

**Affiliations:** 1 Department of Pharmaceutics, School of Pharmacy, China Medical University, Liaoning 110122, China; sally00125@163.com; 2 Key Laboratory of Reproductive Health, Liaoning Research Institute of Family Planning, Liaoning 110031, China; yanglq@lnszjk.com.cn (L.Y.); zhangchong0417@126.com (C.Z.); danhliu@sina.com (D.L.);; 3 Shenyang Institute for Drug Control, Liaoning 110084, China; mengshu888@sina.com

**Keywords:** in situ forming implant, burst release, permeability, solvent exchange, degradation

## Abstract

To explore the mechanism of drug release and depot formation of in situ forming implants (ISFIs), osthole-loaded ISFIs were prepared by dissolving polylactide, poly(lactide-*co*-glycolide), polycaprolactone, or poly(trimethylene carbonate) in different organic solvents, including *N*-methyl-2-pyrrolidone (NMP), dimethyl sulfoxide (DMSO), and triacetin (TA). Drug release, polymer degradation, solvent removal rate and depot microstructure were examined. The burst release effect could be reduced by using solvents exhibit slow forming phase inversion and less permeable polymers. Both the drug burst release and polymer depot microstructure were closely related to the removal rate of organic solvent. Polymers with higher permeability often displayed faster drug and solvent diffusion rates. Due to high polymer-solvent affinity, some of the organic solvent remained in the depot even after the implant was completely formed. The residual of organic solvent could be predicted by solubility parameters. The ISFI showed a lower initial release in vivo than that in vitro. In summary, the effects of different polymers and solvents on drug release and depot formation in ISFI systems were extensively investigated and discussed in this article. The two main factors, polymer permeability and solvent removal rate, were involved in different stages of drug release and depot formation in ISFI systems.

## 1. Introduction

Implants received additional significance as typical controlled release systems in the last decades. However, traditional preformed implants must be administered subcutaneously by a special application device or through a larger needle. Painful administration caused poor compliance of patients. Solvent removal precipitation-based in situ forming implants (ISFIs), developed by Dunn et al, have gained increasing attention and have found a wide variety of practical applications due to their distinct advantages [1,2]. The marketed ISFIs, such as Atridox^®^, showed a significant improvement in patient compliance and therapeutic effect to periodontal disease, and Eligard^®^ for the treatment of prostate cancer [3,4,5,6]. This injectable implant system consists of water-insoluble polymers and organic solvents that are fully or partially water miscible. Polymeric depots are formed through the mechanism of non-solvent-induced phase separation [7]. This mechanism leads to high sensitivity of the ISFI to the properties of the polymer and interactions among the polymer, solvent, and drug [8]. Typically, the drug release from ISFI can be separated into three steps: The initial burst release, then a long period of constant release mainly caused by matrix diffusion and finally the last stage of release due to the erosion of the polymer. In fact, it is not uncommon for the initial burst release of the drug to be seen from solvent removal precipitation-based ISFIs. The initial large bonus of drug can result in tissue irritation and even systemic toxicity, also reduced the effective lifetime of implants. The delay between administration and depot formation has been regarded as the chief cause of burst release [9]. Many methods, such as quantitative ultrasound (QUS), photoacoustic (PA) imaging and UV-vis imaging, have been used to monitor the phase separation of ISFIs [10,11]. Many efforts have been made to the modification of burst release. However, a true solution to reduce drug burst release has not yet been found.

The drug release rate from a polymer matrix is closely related to the polymer properties, such as the molecular weight, molecular structure, crystallinity and degradation rate. ISFI formulations with many biodegradable polymers, such as polylactide (PLA), poly(lactide-*co*-glycolide) (PLGA), or polycaprolactone (PCL), have been investigated [12]. These polymers are crystalline or semicrystalline polymers and degrade by the route of bulk erosion [13]. Drug release from these devices often appears as non-linear profiles with high drug burst release. Aliphatic polycarbonates such as poly(trimethylene carbonate) (PTMC), which is a surface-eroding system, have unique advantages in applications as drug carriers because the rate of degradation is relatively constant and remains steady before full degradation [14,15]. Polymers with different degradation mechanisms and different properties have not been compared in ISFIs in previous studies to investigate the influence of polymers on drug release.

The biocompatible organic solvents used in ISFI can be divided into two major classes [16]. Fast phase inversion (FPI) systems, such as *N*-methyl-2-pyrrolidone (NMP) and dimethyl sulfoxide (DMSO), are highly polar solvents that are readily soluble in water. Slow phase inversion (SPI) systems are usually weak or non-polar solvents, such as triacetin (TA) and benzyl benzoate (BB), which are hardly miscible with water. Different solvent properties result in different phase separation mechanisms, and the polymer matrix and drug release profiles are also different [8,17].

**Figure 1:**
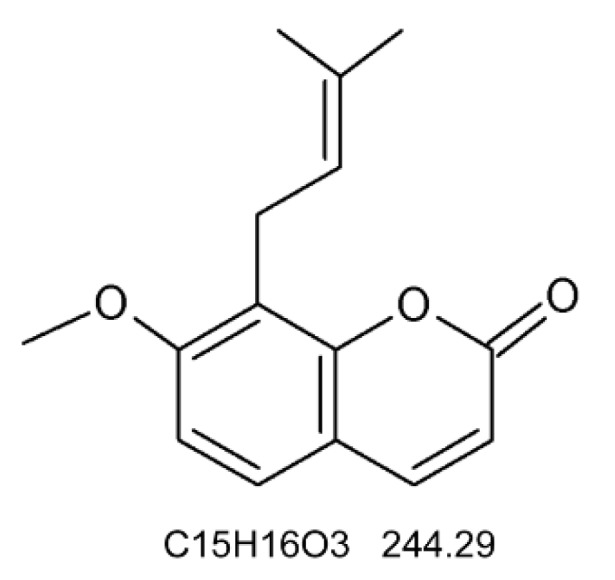
The chemical structure of Osthole.

Previous studies have shown that the physicochemical characteristics of the pharmaceutical composition are equally important factors in drug release, particularly burst release [7]. Drug characteristics, including molecular weight, solubility, lipophilicity, acidity and particle size can alter the release behavior of ISFI. Small molecular weight drugs pass easily through porous structures of implants due to the small size and osmotic pressures [16]. Hydrophobic drugs with high partition coefficients are more easily released and result in high burst release [18]. It is also reported that the strong ionic interaction between basic drug and polymer having terminal carboxyl groups also affected the matrix diffusion process [19]. In this paper, to evaluate the mechanism of drug burst release, natural model drug with low molecular weight should be chosen to get obvious drug burst release effect and avoid influence of drug ionization. As well, hydrophobic drug was selected to investigate the relationship between organic solvent removal and drug release. Osthole (Ost, 7-methoxy-8-isopentenoxycoumarin, Figure 1) is a natural coumarin derivative extracted from *Cnidium monnieri (L.)* Cusson. Many previous studies have confirmed the pharmacological effects of Ost [20], including anti-fibrotic, anti-inflammatory, anti-apoptotic, anti-scratching, free radical scavenging, urination promoting and tranquilizing activities [21]. However, the properties of Ost, such as its water insolubility, poor oral absorbability and photodecomposition, limit its clinical application [22]. Ost was used as a typical natural, small-molecule and hydrophobic drug to investigate the drug release in ISFIs with different compositions.

For the first time, a systemic comparison of ISFIs prepared with different polymers, including PCL, PLA, PLGA and PTMC, and with different biocompatible organic solvents, such as NMP, DMSO and TA, was performed in this paper. In addition, polymer degradation, solvent removal rate and depot morphology were analysed to illustrate the mechanisms of drug release and depot formation. 

## 2. Materials and Methods

### 2.1. Materials

Osthole (Ost) was purchased from Xi’an xiaocao Pharmaceutical Ltd (Xi’an, China). PLA (weight average molecular weight, Mw = 4.75 kDa), PLGA (lactide/glycolide ratio of 50:50, Mw = 5.52 kDa), PCL (Mw = 5.74 kDa) and PTMC (Mw = 5.21 kDa) were procured from Jinan Daigang Biomaterial Co., Ltd (Jinan, China). The organic solvents NMP, DMSO and TA, all of which were of analytical grade, were purchased from Sinopharm Chemical Reagent Co., Ltd (Shanghai, China). HPLC grade acetonitrile, methanol and tetrahydrofuran were obtained from Fisher Chemical Co. Inc. (Geel, Belgium). Polyethylene glycol PEG-35 castor oil (Cremophor^®^ EL) was produced by BASF (Ludwigshafen, Germany). Water was purified with a Milli-Q system (Millipore, USA). All other chemicals used were of analytical grade or of the highest purity and were employed without any additional purification.

Sprague–Dawley rats, weighing 180–220 g, were provided by Liaoning Changsheng Bio-Tech Co., Ltd. (Benxi, China). The experimental protocol was approved by Ethics Committee of Liaoning Research Institute of Family Planning (project identification code 2016-012, approved in June 2016). All measures taken for the rats were performed in accordance with the Regulations of Experimental Animal Administration issued by the people’s Government of Liaoning province (Decree No. 143 of 1 October 2002).

### 2.2. Preparation of Ost-Loaded ISFIs

An appropriate amount of polymer was dissolved in organic solvent to prepare a 15% (*w*/*w*) polymer solution. The polymer solution was stirred for 24 h and kept at room temperature (20–27 °C) until it was clear and transparent. Then, gradually stirring in of an appropriate amount of Ost was performed until complete dissolution to a final concentration of 40 mg/mL.

### 2.3. In Vitro Drug Release and Polymer Degradation

0.5 g Ost-loaded ISFI was injected through a syringe with a 21 gauge needle into 20 mL of a 0.05 M PBS bath (pH 7.4; 37 °C) containing 5% (*w*/*v*) Cremophor^®^ EL to increase the solubility of the Ost and maintain sink conditions. The syringe was weighed before and after injection to calculate the dosage of Ost participating in the experiment accurately. The samples were then incubated in an incubator shaker (HNY-1102C, TianJin Honour Instrument Co., Ltd, Tianjing, China) set at 37 °C and 65 rpm. At specified time intervals, 20 mL of the release medium was replaced by fresh media.

A Nexera UHPLC system (Shimadzu, Milano, Italy) consisting of an LC-30AD pump, an SIL-30AC autosampler, an SPD-M20A photodiode array detector and a CTO-30A column oven was used to measure the Ost content, and the detection wavelength was set at 322 nm. A Shim-pack GIST C18 column (50 mm × 2.1 mm, 2 μm) was used for the separation. Methanol and water (71: 29, % *v*/*v*) were used as the mobile phase, and the flow rate was 0.2 mL/min. The column temperature and injection volume were 35 °C and 10 μL, respectively.

The molecular weights of the retrieved implants were also tested at the appropriate time to evaluate the degradation of the polymer. The retrieved samples were freeze-dried (Alpha 1-2 LD Freeze dryer, Christ, UK) before analysis. The number average molecular weight (Mn), weight average molecular weight (Mw) and polydispersity index (PDI = Mw/Mn) were determined by gel permeation chromatography (GPC) on a Waters 1515 system at 30 °C using THF as the eluent (1 mL/min).

### 2.4. In Vitro Solvent Removal Rate from ISFI

The amount of NMP, DMSO and TA released from the ISFI into the release medium was measured by HPLC methods as mentioned in previous literature reports [23,24]. The solvent removal experimental method, equipment and release medium were the same as those in the in vitro drug release study in Section 2.3. Briefly speaking, 0.5 g blank ISFI solution was injected into 20 mL of release medium and incubated. After 5 h, 1 days, 2 days and 4 days, 20 mL of the release solvent was replaced by fresh medium, and the amounts of NMP, DMSO and TA were measured by UHPLC. The chromatographic analysis conditions are as follows: the mobile phase for DMSO was 10% methanol in water, and the detector was set at 214 nm [25]. For NMP, a 15% acetonitrile solution containing 0.1% trifluoroacetic acid (TFA) was used as the elution solvent, and the UV detection wavelength was 220 nm [26]. The optimized mobile phase for TA consisted of 32% citric acid-hydrochloric acid-sodium hydrate buffer (pH 3.0), 30% acetonitrile and 38% methanol [27]. The detector was set at 215 nm.

### 2.5. Polymer-Solvent Affinity Evaluation

Hildebrand’s one-component and Hansen’s multicomponent solubility parameters were used to estimate the polymer-solvent affinity [28]. Hildebrand’s parameter δ is introduced from the enthalpy of vaporization and can represent the dissolvability of a polymer in different solvents. Hansen suggested that Hildebrand’s solubility parameter δ can be divided into three different cohesive forces: non-polar dispersion forces (δ_d_), polar dispersion forces (δ_p_) and hydrogen bonding (δ_h_) (Equation (1)) [29].

(1) $$\delta ={\left(\delta_{d}^{2}+\delta_{p}^{2}+\delta_{h}^{2}\right)}^{1/2}$$

The solvent and solute have similar solubility parameters, indicating that there is high affinity between them and that they are easily dissolved by each other [8]. The comparison Δδ (Equation (2)) can be used to predict the affinity between solvent and polymer [29,30].

(2) $$\Delta \delta ={\left[4{\left(\delta_{s}-\delta_{p}\right)}_{d}^{2}+{\left(\delta_{s}-\delta_{p}\right)}_{p}^{2}+{\left(\delta_{s}-\delta_{p}\right)}_{h}^{2}\right]}^{1/2}$$

where δ_s_ is Hansen’s solubility parameter of the solvent and δ_p_ is Hansen’s solubility parameter of the polymer.

### 2.6. Morphological Analysis of ISFI Depots

The inner/surface morphologies of completely solidified implants were examined using a VEGA3 TESCAN (TESCAN, Brno, Czech Republic) scanning electron microscope (SEM). After rinsing in water carefully and then freeze-drying (Alpha 1-2 LD Freeze dryer, Christ, UK), the samples were treated with liquid nitrogen and then crushed. Samples were relocated onto metal stubs by double-adhesive conductive tape and sputter-coated with platinum using an EMITECH SC7620 sputter coater (Quorum Technologies Ltd., Laughton, United Kingdom). The voltage was set to 3.0 kV for SEM observation.

### 2.7. In Vivo Drug Release Study

The formulation for the in vivo release study was prepared by strict aseptic technology. After acclimation for at least one week before the studies, Sprague-Dawley rats were weighed and randomly divided into a study group and a control group. Then, the rats were anaesthetized using isoflurane on the day of the experiment. Aseptic Ost-loaded ISFI (290.4 ± 21.7 mg) was injected into the midline dorsal area under the skin using a 21 gauge needle. The control group was treated with blank ISFI formulation.

At predetermined time points, five rats in each group were selected at random, and blood samples (approximately 0.7 mL) were collected from the lateral tail vein into heparin-containing tubes. Then, plasma was immediately separated by centrifugation at 3500 rpm for 10 min and frozen at −20 °C for subsequent analysis. Next, the rats were sacrificed, and the residual drug content was detected by retrieving the implants. The retrieved implants were shred, then extracted in 50 mL of methanol and sonicated for 2 h. After filtration, the Ost concentration was determined by UHPLC as mentioned in 2.3.

Ten microliters of imperatorin in methanol (7.95 μg/mL) was added to 200 μL of a plasma sample as an internal standard and vortexed for 1 min. Then, the mixture was extracted with 1 mL of ether. The extraction was transferred to another centrifuge tube and dried with nitrogen. Finally, the residue was dissolved in 75 μL of mobile phase for analysis by UHPLC as mentioned in Section 2.3.

### 2.8. Statistical Analysis

The data obtained were analysed by one-way analysis of variance ANOVA using the SPSS version 19.0 (SPSS Inc., Chicago, IL, USA) for Windows software package. Significant differences among groups were determined when *P < 0.05*.

## 3. Results

### 3.1. In Vitro Ost Release and Polymer Degradation

**Figure 2:**
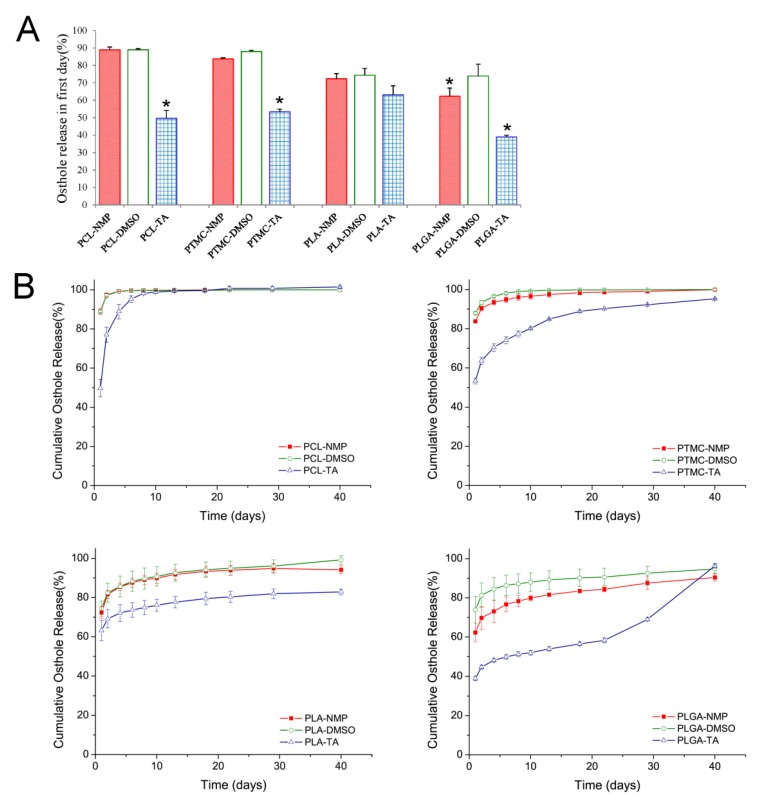
In vitro release of Osthole from ISFIs prepared by different polymers and solvents. (**A**) The burst release effect. (**B**) In vitro release profiles. (mean ± S.D., *n* = 5). *P < 0.05.

The Ost release profiles of formulations composed of different polymers and solvents are presented in Figure 2.

All formulations showed high burst release effects, especially the formulations containing NMP and DMSO. On the first day, PCL based formulations with NMP and DMSO as organic solvents presented the highest initial burst effect, and more than 88% of Ost had been released. The slowest initial drug release was found in the PLGA based ISFI with TA as the organic solvent, which released 38.92% of Ost on the first day. After burst release, the release of Ost was still fast in PCL and PTMC based ISFIs, and the drugs was totally released before the end of 40 days. However, in PLA and PLGA contained implants, the release of drugs was relatively slow. However, after day 22, the release of Ost sped up dramatically in PLGA ISFIs and the Ost was close to complete release on day 40.

**Figure 3:**
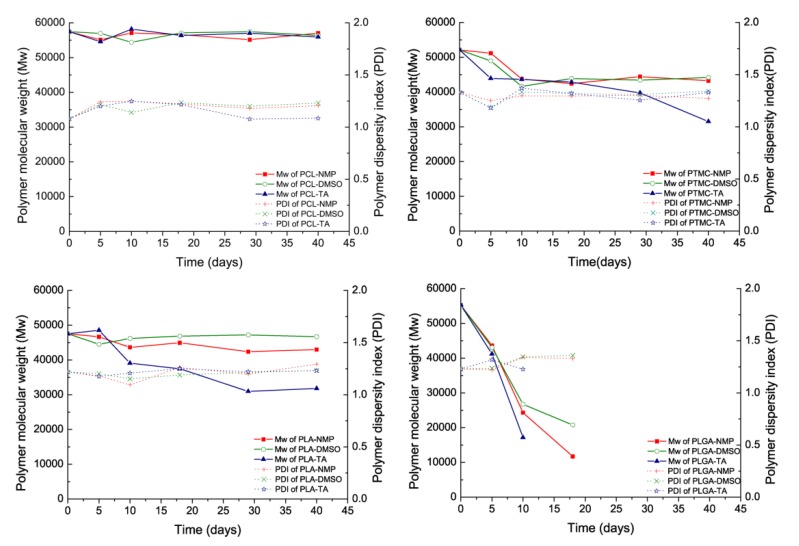
The changes in the molecular weight (Mw) of the ISFIs as well in the polydispersity index (PDI) (Mw/Mn). (mean ± S.D., *n* = 3).

Both the diffusion of Ost and degradation of the polymer contributed to the release when the polymer began to degrade. As shown in Figure 3, PCL and PTMC degradation were markedly slower than those of the other polymers. However, the molecular weight of PLGA was lost very quickly and could not be accurately tested after 15 days. In addition, it should be noted that there was an acceleration in polymer degradation when using TA as the organic solvent, which has never previously been mentioned or discussed.

### 3.2. Solvent Removal Rate from ISFI

**Figure 4:**
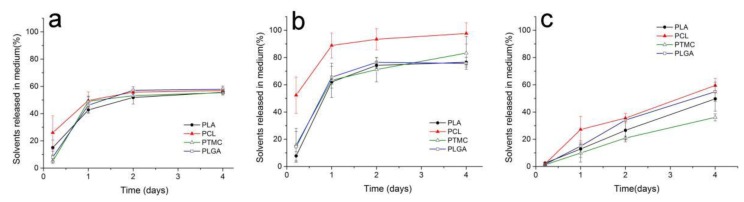
The removal rate of organic solvents (**a**) NMP, (**b**) DMSO and (**c**) TA from different polymer ISFIs. (mean ± S.D., *n* = 3).

Figure 4 shows the solvent released into the release medium from different ISFI depots. The removal rate of TA was obviously slower than that of DMSO and NMP. On the first day, 46.8% of NMP and 69.9% of DMSO were released averagely, while only 16.2% of TA was exchanged. In addition, the solvent exchange of NMP and DMSO was not completely. After the rapid exchange in the first few days, approximately 45% of NMP remained in all depots, and more than 17% of DMSO remained in all the polymer depots except PCL.

### 3.3. Polymer-Solvent Affinity Evaluation

Hildebrand’s one-component and Hansen’s multicomponent solubility parameters were used to explain the difference in solvent exchange rate and the phenomenon of the remaining solvent. Table 1 shows the solvent and polymer solubility parameter data published in the literature [31,32,33,34,35].

The comparison Δδ was calculated using Equation (2) to predict the affinity between solvent and polymer. The comparison Δδ values are given in Table 2. A low Δδ indicated high affinity between the organic solvent and polymer [8].

### 3.4. Morphological Analysis of ISFI Depots

**Figure 5:**
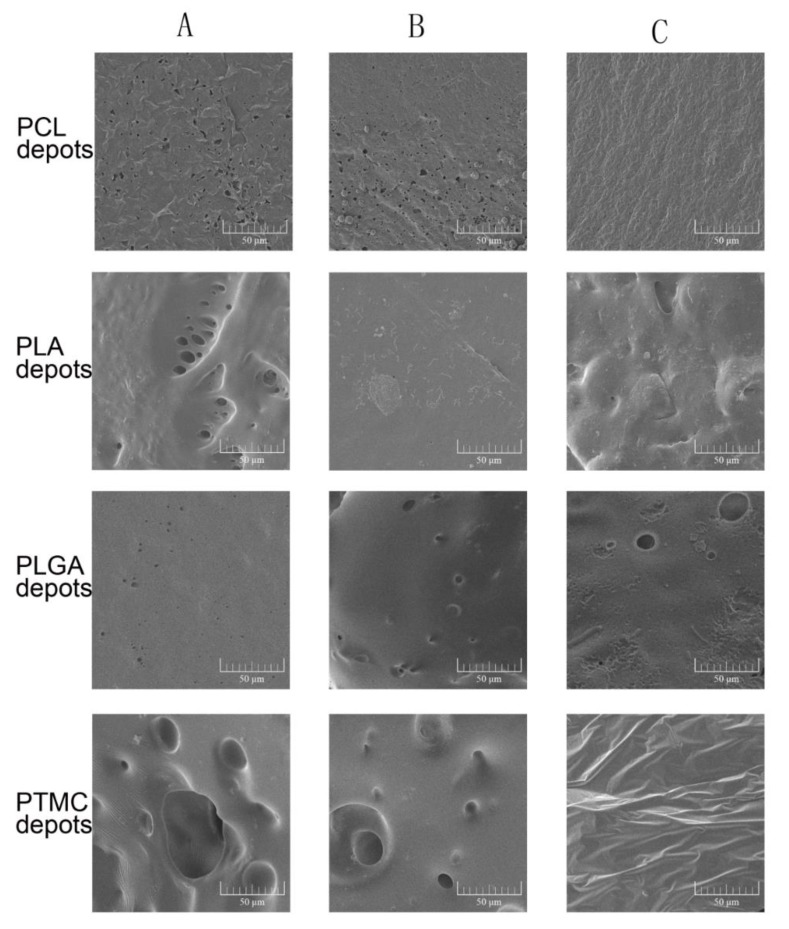
The surface morphologies of different ISFI depots examined by scanning electron microscopy. (**A**) ISFI depots prepared by 15% DMSO; (**B**) ISFI depots prepared by 15% NMP; (**C**) ISFI depots prepared by 15% TA.

**Figure 6:**
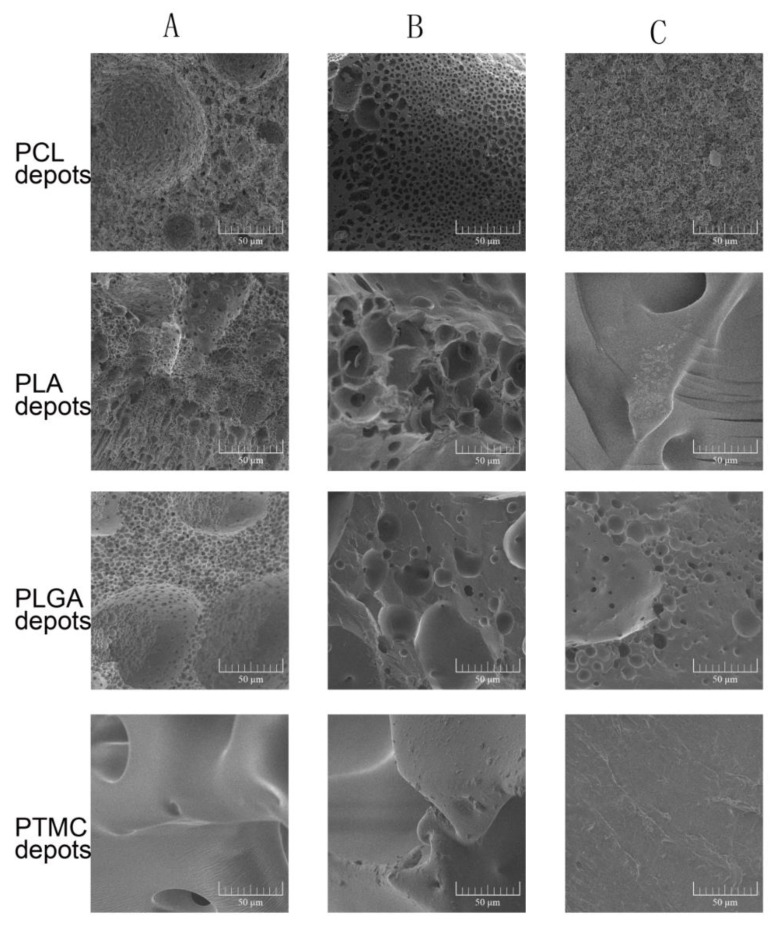
The cross-section morphologies of different ISFI depots examined by scanning electron microscopy. (**A**) ISFI depots prepared by 15% DMSO; (**B**) ISFI depots prepared by 15% NMP; (**C**) ISFI depots prepared by 15% TA.

The ISFIs containing DMSO and NMP formed porous, soft and plastic foam-like implants quickly. However, the formation of TA contained ISFIs spent several days, and the formed depots were dense and hard. Figure 5 shows the surface morphologies of different ISFIs. The surface structures of the TA-induced depots were dense, and few bubbles were observed. When hydrophilic solvents such as DMSO and NMP were used, the surface of the depots were full of holes. The observations of the inner structure were consistent with the porosity of the surfaces (Figure 6). An interesting finding was that the PTMC depots formed with DMSO or NMP were flexible, porous, rigid colloid structures, whose holes were much larger than the other ISFIs. However, the PTMC depots lacked any tiny pores when examined under magnification.

### 3.5. Drug Release of Ost-ISFI In Vivo

**Figure 7:**
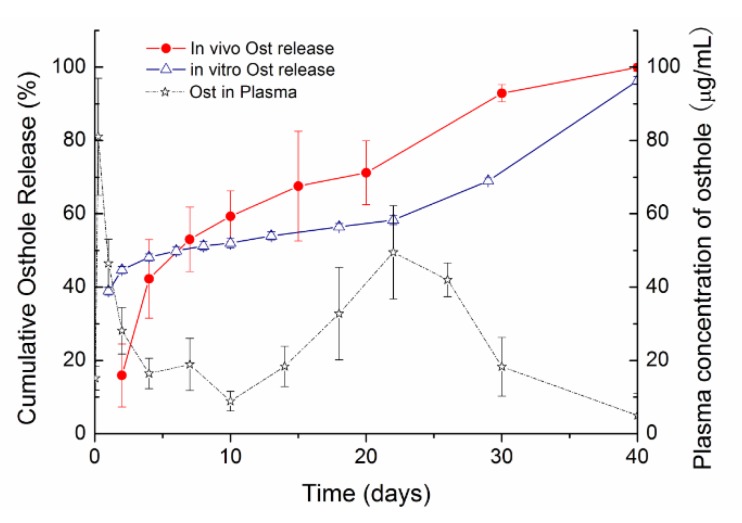
Osthole release from ISFI in vitro and in vivo, and plasma concentrations of Osthole after the administration. (mean ± S.D., *n* = 3–5).

To investigate the release mechanism of the ISFI system under physiological conditions, the in vitro and in vivo drug release profiles of the ISFI prepared with PLGA and TA are compared in Figure 7. In vivo, the initial release was significantly reduced (15.9% until 2 days), then much faster drug release was observed (*p* < 0.05).

After subcutaneous injection of Ost-ISFI, the Ost plasma concentration presented a double peak curve. Ost-ISFI maintained the plasma Ost concentration over 30 days, and the two maximum concentrations (C_max_) achieved in serum were 81.0 and 49.5 μg/mL, which were reached at 6 h and 22 days after administration, respectively. The lowest concentration over 30 days was 8.9 μg/mL, which was detected on day 10.

## 4. Discussion

The release of drug from an ISFI is a complex process involving a series of steps, such as solvent exchange, polymer precipitation, depot formation, drug diffusion and polymer degradation. All of the steps could be affected by the physical and chemical properties of the polymer, the organic solvent, the drug, and the interactions among them. Drug release could be separated into three steps: the initial burst release, then a long period of constant release mainly caused by matrix diffusion and finally the last stage of release due to the erosion of the polymer [23].

Ost is a typical small-molecule and highly lipid-soluble drug. According to our research, the burst release effects of Ost were quite high in all formulations, especially the ISFIs containing NMP and DMSO. The small molecular size of Ost was probably the main reason of high burst release effect [18]. During depot formation, Ost could easily transfer into aqueous medium with organic solvent, even prior to being completely encapsulated. So, the burst release effect of the drug was also deeply influenced by the solvent properties. Solvent that had a high-water affinity exhibited obviously burst release of drug. For example, the burst release in formulations containing TA was significantly lower than that in formulations containing NMP and DMSO (Figure 2A and Figure 4). Camargo et al. studied the effect of several biocompatible solvents on the release of ivermectin(IVM) from PLA formed ISFIs [8]. The same conclusion was obtained that the release rate of IVM increased with increasing water miscibility of the solvent. 

After burst release, the constant drug release was recognized as a stage caused by matrix diffusion. As well, our results indicated that polymer permeability was the main factor to affect drug release in this stage. PCL was reported to be highly permeable to several drugs, whereas PLA was found to be 10^4^ times less permeable than PCL [36,37]. PTMC is an amorphous and non-crystalline polymer with more free volume to increase the permeability. Thus, although the glass temperature (T_g_) of PTMC (−26−15 °C) was higher than that of PCL (−60 °C), which could reduce the permeability, the diffusion coefficient of PTMC was reported to be similar to that of PCL [38]. Regarding PLGA, the proportion of copolymer monomers had a great effect on T_g_ and crystallinity [39]. The random copolymerization of glycolic acid and lactic acid decreased the crystalline degree of PLGA but increased the rate of hydration and hydrolysis. Therefore, the progesterone diffusion rate was slightly higher in PLGA than in PLA, especially when PLGA contained a high proportion of glycolic acid [40]. As shown in Figure 2B and Figure 4, polymers with high permeability displayed faster drug release and more rapid solvent exchange. These results demonstrated that polymer permeability played a significant role in the release of both drugs and organic solvents [41], and confirmed the diffusion mechanism of the drug and the importance of polymer permeability to drug diffusion.

As shown in Figure 3, an interesting result was that the solvent exchange was not completely, and part of solvents could remain in the formed depots. Such a result indicated that the solvent exchange process was not only affected by the polymer permeability and water solubility of the solvent. Some researchers have suggested that the remaining solvents may be caused by the lower water absorption of the polymer and high solvent–polymer affinity, but this hypothesis has not yet been confirmed [24]. In this paper, the solvent–polymer affinity was estimated by Hansen’s solubility parameters according to the popular method as Equations (1) and (2) [30,42]. When the solubility parameters of the solvent and polymer were similar, a low Δδ could be obtained, which suggested that the polymer-solvent affinity was high. As shown in Table 2 and Figure 4, there were low Δδ values between the remaining solvents and polymers. Meanwhile, high Δδ values corresponded to quick solvent exchange rates. The results indicated that both polymer permeability and polymer-solvent affinity influenced the process of solvent exchange. 

It is well known that both drug diffusion and polymer degradation contribute to drug release when the polymer begins to degrade. PCL and PTMC degradation were markedly slower than the other polymers, and the drug release rate remained unchanged at the end of the release period. This result was the same as those in previous literature reports [14,43,44]. However was much more quickly and finally caused the disintegration of the implants. As a result, accelerated and complete release of Ost occurred in PLGA ISFIs after day 22 (Figure 2). The release profile of Ost from PLGA and TA based ISFI showed typical tri-phasic release as summarized by Fredenberg et al [45,46]. Phase I was the burst release, phase II was a release plateau described as drug diffusion phase and finally phase III named degradation-facilitated release, sometimes also called the second burst. According to our investigation, the process of polymer degradation was also influenced by the organic solvent. TA often led to a dense polymer-rich depot, which could reduce the moisture content of the depots and thus decelerate the polymer degradation. However, there was a significant acceleration in polymer degradation when using TA as the organic solvent (Figure 3), which has never previously been mentioned or discussed. TA is a triacetate ester of glycerol that produces acetic acid during its hydrolysis [47]. Ester hydrolysis could be accelerated by acid-base catalysis, so the increasing concentration of acetic acid was expected to speed up the hydrolysis of the polymer and of TA itself. Such a process is often said to be autocatalytic [43]. The autocatalytic effect should be considered when using TA as an organic solvent, especially when the loaded drug was a readily hydrolyzed material.

The in vivo release of Ost from PLGA based ISFI was shown in Figure 7. As discussed above, the first peak concentration was due to the burst release of Ost, and the second peak concentration was caused by the breakup of the depots after polymer degradation. The bimodal curve of Ost plasma concentration was consistent with the in vitro release behavior of Ost. However, unlike the simple release medium in vitro, there were many more factors influencing the release kinetics in vivo. After subcutaneous injection, both the initial drug release and depot formation were delayed because of insufficient body fluid [48]. As well, because of the enzymatic, mechanical and chemical effects in the complex physiological environment, the biodegradation process of the polymer was much faster in vivo than in vitro. 

In this paper, the formation of the ISFI depots were also investigated. As to the results, the solvents removal rate not only played an important role in the release of the drug, especially the initial burst release, but also governed the formation and final state of the ISFI depots [24]. It is generally recognized that FPI solvents are more likely to develop loose and porous structures [49]. As shown in Figure 5 and Figure 6, TA led to a dense polymer-rich depot with minimal drug burst release, while NMP and DMSO based ISFIs formed a heterogeneous and porous polymer matrix with higher burst release. As well, the porosity of the depots was closely related to the solvent exchange rate. Quickly exchange of organic solvent caused spongy depots with more pores, while ISFI with slow solvent removal rate was compact or non-porous. In addition, our research showed the polymer properties also affected the final state of depots. PTMC is an amorphous material with a low glass transition temperature of approximately –20 °C [50]. Unlike semi-crystalline polymers, PTMC shows rubbery state under physiological temperature. So, the precipitated PTMC was in an unstable state during depot formation, and the tiny pores were easily destroyed to form larger ones. As a result, the final depots of PTMC formed with DMSO or NMP were rigid colloid sponge-like structures with large holes but without any tiny pores (Figure 5 and Figure 6). 

## 5. Conclusions

In this study, ISFIs were prepared with different polymers and solvents. The effects of polymer properties and solvent removal rate on implant formation and the release of Ost were evaluated. The burst release effect of the drug was deeply influenced by the solvent removal process. During phase inversion, Ost transfer into aqueous medium with organic solvent before being completely encapsulated by precipitated polymer. The burst release effect of Ost could be reduced by using hydrophobic solvent TA and less permeable polymers, such as PLGA and PLA. Polymers with higher permeability often displayed faster drug and solvent diffusion rates, which indicated the diffusion mechanism of ISFI. Both the drug burst release and polymer depot morphology are closely related to the solvent release rate. Quickly solvent removal tends to develop loose and porous structures. When considering the solvent exchange process, solvent water solubility, polymer permeability and polymer-solvent affinity should be taken into account. Due to high polymer-solvent affinity, some of the organic solvent remained in the depot even after the implant was completely formed. In addition, some interesting findings were also made in this article. For example, due to the unique mechanical properties of PTMC, the depot formed by PTMC showed an unusual structure without tiny pores. We also found that polymer degradation could be accelerated when using TA as the solvent. The autocatalytic effect of TA should be considered when loading hydrolysable drugs.

In summary, the effects of polymer and solvent properties on drug release and depot formation in ISFI systems have been investigated and discussed extensively in this article. The results in this article will undoubtedly promote research and development in the concerned area.

## Figures and Tables

**Table 1:** Solubility parameters of solvents and polymers.

Solvents & Polymers	Hildebrand’ (cal/cm^3^)^½^	Hansen’s Solubility Parameters (MPa^1/2^)		
	*δ*	*δ*_d_	δ_p_	δ_h_
NMP^a^	22.9	18.0	12.3	7.2
DMSO^a^	19.4	18.4	16.4	10.2
TA^a^	26.7	16.5	4.5	9.1
PLA^b^	21.7	18.5	9.7	6.0
PCL^c^	19.7	17.7	6.2	7.8
PLGA^d^	22.3	17.4	9.1	10.5
PTMC^e^	20.2	15.3	7.4	10.8
Water^a^	47.8	15.5	16	42.3

^a^ From [31], ^b^ From [32], ^c^ From [33],^d^ From [34], ^e^ Calculated by the van Krevelen group contribution optimization method [35].

**Table 2:** The comparison Δδ between solvents and polymers.

Solvents	Δδ Solvent/Polymer (MPa^1/2^)			
	PLA	PCL	PLGA	PTMC
NMP	2.9	6.2	4.8	8.2
DMSO	7.7	10.6	7.6	11.0
TA	7.5	3.2	5.1	4.1
